# Current situation of complications related to reconstructive surgery for pelvic organ prolapse: a multicenter study

**DOI:** 10.1007/s00192-021-04892-x

**Published:** 2021-06-24

**Authors:** Zhi-jing Sun, Tao Guo, Xiu-qi Wang, Jing-he Lang, Tao Xu, Lan Zhu

**Affiliations:** 1 Department of Obstetrics and Gynecology, Peking Union Medical College Hospital, Peking Union Medical College, Chinese Academy of Medical Sciences, National Clinical Research Center for Obstetirc & Gynecologic Diseases, Beijing, China; 2 Department of Epidemiology and Biostatistics, Institute of Basic Medical Sciences, Chinese Academy of Medical Sciences & School of Basic Medicine, Peking Union Medical College, Beijing, China

**Keywords:** Pelvic organ prolapses, Transvaginal mesh, Intraoperative complications, Postoperative complications

## Abstract

**Introduction and hypothesis:**

This study aimed to investigate the evaluation and management of complications after pelvic floor reconstructive surgery for pelvic organ prolapse in China.

**Methods:**

Complications of pelvic floor reconstructive surgery for pelvic organ prolapses from 27 institutions were reported from November 2017 to October 2019. All complications were coded according to the category-time-site system proposed by the International Urogynecological Association (IUGA) and the International Continence Society (ICS). The severity of the complications was graded by the Clavien-Dindo grading system. Four scales were used to evaluate patient satisfaction and quality of life after management of the complications: the Patient Global Impression of Improvement (PGI-I), the Pelvic Floor Impact Questionnaire Short Form (PFIQ-7), the Pelvic Organ Prolapse Symptom Score (POP-SS), and a 5-point Likert-type scale that evaluated the patient’s choice of surgery.

**Results:**

Totally, 256 cases were reported. The occurrence of complications related to transvaginal mesh (TVM) and laparoscopic sacrocolpopexy (LSC) had a significantly longer post-surgery delay than those of native tissue repair surgery (*p* < 0.001 and *p* = 0.010, respectively). Both PFIQ-7 and POP-SS score were lower after management of complications (*p* < 0.001). Most respondents (81.67%) selected very much better, much better, or a little better on the PGI-I scale. Only 13.3% respondents selected unlikely or highly unlikely on the 5-point Likert-type scale.

**Conclusions:**

The occurrence of complications related to TVM surgery and LSC had a longer post-surgery delay than native tissue repair surgery. Long-term regular follow-up was vital in complication management. Patient satisfaction with the management of TVM complications was acceptable.

## Introduction

Surgery is an important method for treating symptomatic pelvic organ prolapses (POP), which is caused by the dysfunction of pelvic floor supporting tissue. For women, the lifetime risk of undergoing surgery for POP is 11.8–12.6% [1, 2]. Synthetic mesh surgeries including transvaginal mesh (TVM) implantation and laparoscopic sacrocolpopexy (LSC) have been common procedures in POP surgeries. Synthetic mesh surgery contributes to a better control of bulge symptoms and better anatomic repair [3, 4]. The recurrence rate of POP for patients undergoing synthetic mesh surgery is also lower than for native tissue repair surgery [5]. However, the use of synthetic mesh simultaneously causes a series of complications, raising wide concerned. Mesh exposure was the most common complication after TVM surgery. It occurred in 1.4–19% TVM surgeries, and 3–8% patients underwent a reintervention for mesh complications after receiving TVM surgery [3]. Perioperative complications, including longer surgery time, greater blood loss, and more bladder perforations, were also more common in TVM than native repair surgery [6, 7]. Claims by women with mesh complications reached over 1 billion dollars [8].

Due to the increasing reports on TVM complications, the US Food and Drug Administration (FDA) issued two warnings in 2008 and 2011. Finally, in April 2019, because of the lack of evidence supporting TVM surgery repair for POP, the FDA ordered all manufacturers to stop selling and distributing TVM designed for POP repair surgery. Then, worldwide, many organizations and associations issued similar statements calling for stopping clinical use of TVM [9].

The use of TVM for POP surgery decreased after the FDA warning in 2011, and it is now banned, but the implantation of mesh had been completed in numerous patients. These patients might suffer from mesh-related complications in the future. However, no standard management of mesh complications is available now [10]. It is important to prospectively follow-up and register the patients who received synthetic mesh surgeries, which would help to establish standard managements for mesh complications, and explore the potential patients who would benefit from synthetic mesh surgery to repair POP.

To investigate the evaluation and management of complications after reconstructive surgery, in China, the Chinese Urogynecological Association launched a report on reconstructive surgery complications in November 2017. The aim of this study was to report the current situation for the complications related to reconstructive surgery for POP in China and to evaluate the management of the complications.

## Materials and methods

### Data collection

The Chinese Urogynecological Association consists of members from 27 academic medical institutions nationwide. These 27 institutions are all tertiary hospitals representing the higher level in the field of pelvic floor reconstruction in China. The study was approved by the institutional ethics committee of the individual institution and registered on the public domain (https://register.clinicaltrials.gov) (registration number: NCT03617211). From November 2017, 27 members agreed to report and register the patients who experienced complications related to reconstructive surgery for POP. Patients who met the following criteria were to be reported and registered: (1) patients with perioperative complications including infection, hematoma, bladder injury, bowel injury, urinary retention, and other complications possibly related to reconstructive surgery; (2) patients with symptomatic or asymptomatic postoperative complications in regular postoperative follow-up; (3) patients referred from other hospitals for POP surgery complications.

In this study, we analyzed data collected between November 2017 and October 2019. We used the terminology laid out by the International Urogynecological Association (IUGA), International Continence Society (ICS), and American Urogynecological Society (AUGS) [10, 11].

### Category-time-site system

The category-time-site (CTS) system was used in this study to classify all complications. The CTS system was proposed by the IUGA and ICS in 2011 [11]. All complications were classified with a code according to three aspects including category, time, and site.

### Clavien-Dindo grading system

We adopted the Clavien-Dindo grading system to evaluate the severity of all complications [12].

### The 5-point Likert-type scale

At the last follow-up, patients with TVM surgery complications were given the question: “If you now had an opportunity to choose again, to what extent would you still choose this type of surgical procedure?” We used a 5-point Likert-type scale to investigate the patients’ impression of the benefits and risk of the TVM surgery after experiencing those mesh complications. Five options were provided including highly likely, likely, equivocal, unlikely, and highly unlikely.

### Patient global impression of improvement scale

Patients with TVM surgery complications completed the Patient Global Impression of Improvement (PGI-I) scale at the last follow-up to evaluate the outcome of the management of complications [13]. Patients were provided with seven options to describe how complications changed compared with the condition before management: very much better, much better, a little better, no change, a little worse, much worse, and very much worse.

### Pelvic organ prolapse symptom score

The Pelvic Organ Prolapse Symptom Score (POP-SS) consisted of seven questions with a total score ranging from 0 to 28 points [14]. A higher score means more severe symptoms. POP-SS was adopted to evaluate the POP symptoms before and after management of TVM surgery complications.

### Pelvic floor impact questionnaire short form

After pelvic surgeries, patients sometimes experienced symptoms or abnormal conditions of the bladder, bowel, or vagina, which often affected the quality of life significantly. The Pelvic Floor Impact Questionnaire Short Form (PFIQ-7) consists of 21 questions, and the validated Chinese version was adopted in this study [15, 16]. We sought to investigate the extent to which their activities, relationships, and feelings had been affected by pelvic floor symptoms aroused by complications and whether complication management improved the symptoms. A higher score means more severe symptoms. Patients with TVM surgery complications completed the PFIQ-7 scale before and after complication management.

### Statistical analysis

Statistical analysis was performed by SPSS software (version 22.0 for Windows, IBM Corp, Armonk, NY, USA). Figures were established by GraphPad Prism (version 8.0.1 for Windows; La Jolla, CA, USA). A two-tailed *p* value < 0.05 was considered statistically significant. The delay of occurrence of complications after initial surgery among TVM surgery and LSC and native tissue repair (NTR) surgery was compared using *t*-test. Management for different complication was compared using Pearson’s chi-square test and Fisher’s exact test. Paired samples Wilcoxon symbol rank test was adopted to compare POP-SS and PFIQ-7 before and after complication management.

## Results

Two hundred fifty-six patients with POP surgery complications were reported from 27 institutions during the 2 years from November 2017 to October 2019. As shown in Table 1, the median age at the time of receiving management for complications was 64 (range 44–69) years. Forty-eight patients (18.8%) were transferred from other hospitals by referral, and 208 patients (82.8%) were managed at the hospital where the initial POP surgery was performed. In the initial surgery, 15 (7.0%) patients underwent native tissue repair (NTR) surgeries, 126 (49.2%) TVM surgeries, 19 (7.4%) TVM plus LSC, 19 (7.4%) TVM plus mid-urethral sling (MUS), 1 (0.4%) TVM plus LSC and MUS, and 76 (29.7%) LSC only (Table 1). One hundred thirty-nine cases reported mesh types. The material of the synthetic mesh was all polypropylene. Twenty-four patients received TiLOOP mesh made by Ethicon Company with a titanium layer.

**Table 1:** Baseline patient characteristics

Parameter	Number of patients	%
Mean age years, (SD)	64.2 (9.8)	
Mean BMI* kg/m^2^, (SD)	25.1 (2.9)	
Median parturition number, (range)	2.0 (1–5)	
Comorbidity		
Diabetes	23	9.0
Hypertension	21	8.2
Chronic cough	12	4.7
Constipation	13	5.1
Other comorbidities	4	1.6
Menstrual status		
Postmenopausal	220	86.3
Premenopausal	36	14.8
Initial surgery category		
NTR	15	7.0
TVM	126	49.2
TVM + LSC	19	7.4
TVM + MUS	19	7.4
TVM + LSC + MUS	1	0.4
LSC	76	29.7
Referral		
No referral	208	82.8
From grade A tertiary hospitals	24	9.4
From other hospitals	24	9.4

*Body mass index, defined as weight in kilograms divided by height in meters squared

SD, standard deviation; BMI, body mass index; NTR, native tissue repair; TVM, transvaginal mesh; LSC, laparoscopic sacrocolpopexy; MUS, mid-urethral sling

**Fig. 1:**
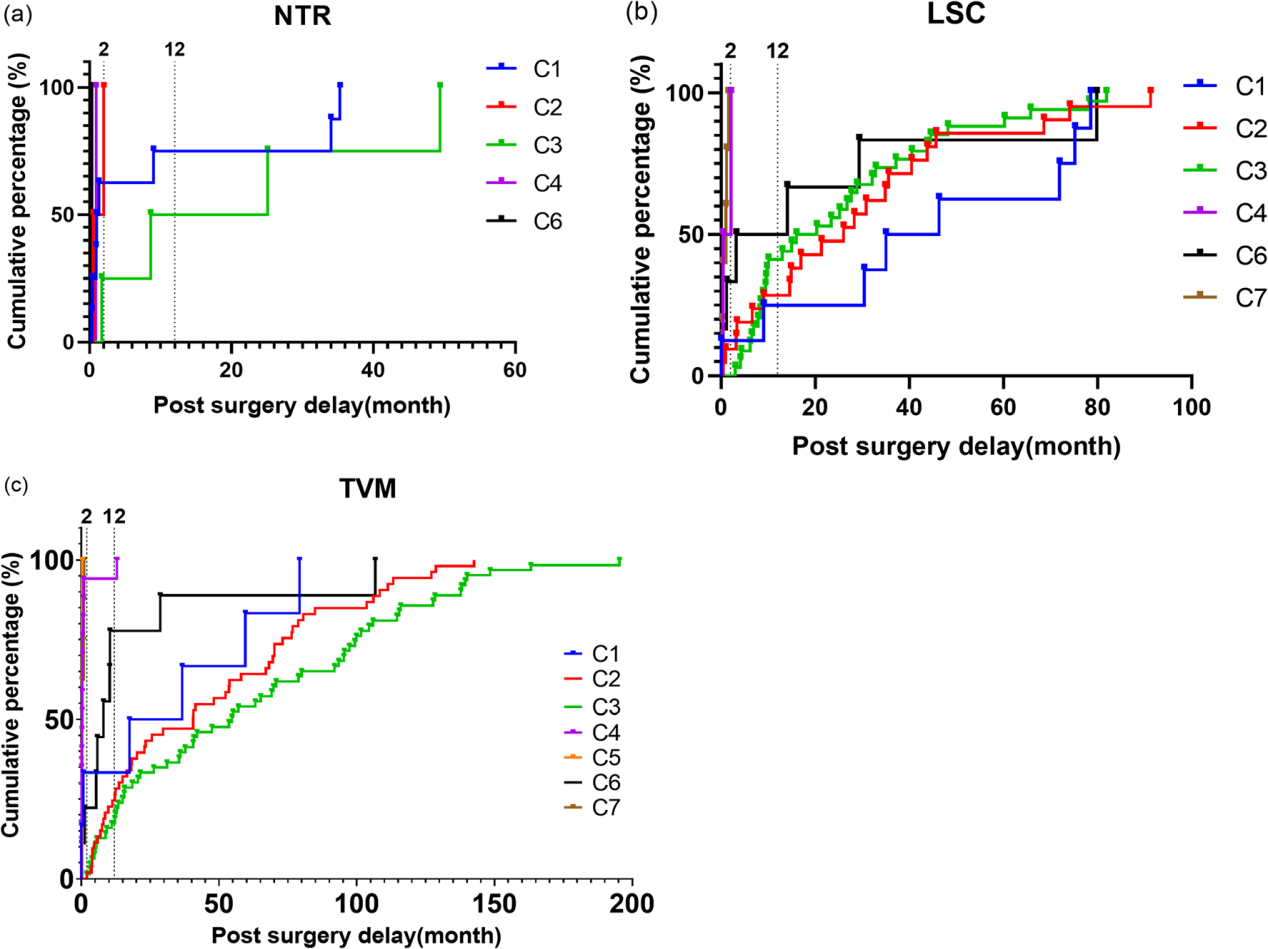
Relation between post-surgery delay and cumulative percentage of complications in native tissue repair (NTR) surgery (a), laparoscopic sacrocolpopexy (LSC) (b), and transvaginal mesh (TVM) surgery (c). C1–C7 refers to category 1–7 in the category-time-site classification system

We coded all the complications according to the CTS system, and the details are presented in Table 2. Categories 2 (30.7%) and 3 (40.3%), which refer to mesh exposure, were the most common complications in synthetic mesh surgery, followed by urinary tract injury (10.0%). Most complications (55.8%) occurred 12 months after synthetic mesh surgery. As shown in Fig. 1, the occurrence of complications related to TVM surgery and LSC had a significantly longer delay than those of NTR surgery (*p* < 0.001 and *p* = 0.010, respectively). Mesh exposure occurred constantly after the initial surgery with a median delay of 44.72 (range 1.93–195.37) months in TVM surgery, longer than the 16.49 (range 0.03–91.23) months for LSC (*p* = 0.003). The longest time span after TVM surgery was 16.28 years. The most frequently involved site was the vagina (76.3%) after synthetic mesh surgery. Furthermore, complications of synthetic mesh surgery in 66.80% patients were asymptomatic or involved no pain. They were identified during regular follow-up.

**Table 2:** Category-time-site classification of all complications reported

		Synthetic mesh surgery			NTR
		TVM ± LSC/MUS	LSC	Total	
Total (*n*)		165	76	241	15
Category (*n*, %)	1: Vagina: no epithelial separation	6 (3.6)	8 (10.5)	14 (5.8)	7 (46.7)
	2: Vagina: smaller epithelial separation/exposure/ulcer ≤ 1 cm	53 (32.1)	21 (27.6)	74 (30.7)	2 (13.3)
	3: Vagina: larger epithelial separation/exposure/extrusion > 1 cm	63 (38.2)	34 (44.7)	97 (40.3)	3 (20.0)
	4: Urinary tract	22 (13.3)	2 (2.6)	24 (10.00)	2 (13.3)
	5: Rectal or bowel	2 (1.2)	0	2 (0.8)	0
	6: Skin and/or musculoskeletal	7 (4.2)	6 (7.9)	13 (5.4)	1 (6.7)
	7: Patient compromise	12 (7.3)	5 (6.6)	17 (7.1)	0
Time (*n*, %)	1: < 48 h	27 (16.4)	4 (5.3)	31 (12.9)	2 (13.3)
	2: 48 h–2 months	16 (9.7)	13 (17.1)	29 (12.0)	9 (60.0)
	3: 2–12 months	30 (18.2)	19 (25.0)	49 (20.3)	1 (6.7)
	4: > 12 months	92 (55.8)	40 (52.6)	132 (54.8)	3 (20.0)
Site (*n*, %)	0: No site applicable	7 (4.2)	6 (7.9)	13 (5.4)	4 (26.7)
	1: Vaginal: area of suture line	118 (71.5)	57 (75.0)	175 (72.6)	8 (53.3)
	2: Vaginal: away from area of suture line	5 (3.0)	4 (5.3)	9 (3.7)	1 (6.7)
	3: Trocar passage/adjoining viscus	27 (16.4)	3 (4.0)	30 (12.5)	0
	4: Other skin or musculoskeletal site	4 (2.4)	2 (2.7)	6 (2.5)	0
	5: Intra-abdominal	4 (2.4)	4 (5.3)	8 (3.3)	2 (13.3)
Pain (*n*, %)	Unspecified	6 (3.6)	1 (1.3)	7 (2.9)	0
	A: Asymptomatic or no pain	114 (69.1)	47 (61.8)	161 (66.8)	12 (80.0)
	B: Provoked pain only	21 (12.7)	12 (15.8)	33 (13.7)	3 (20.0)
	C: Pain during sexual intercourse	6 (3.7)	4 (5.3)	10 (4.2)	0
	D: Pain during physical activities	5 (3.0)	2 (2.6)	7 (2.9)	0
	E: Spontaneous pain	13 (7.9)	10(13.2) (13.2)	23 (9.54)	0

TVM, transvaginal mesh; LSC, laparoscopic sacrocolpopexy; NTR, native tissue repair; MUS, mid-urethral sling

**Fig. 2:**
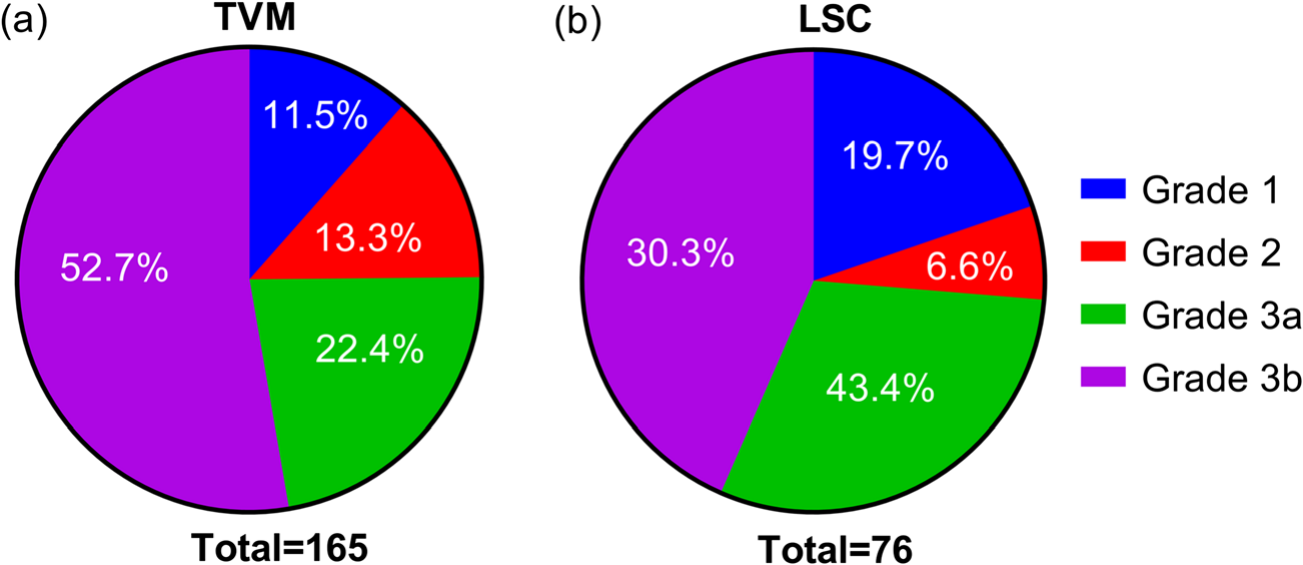
Clavien-Dindo grading of complications in transvaginal mesh (TVM) surgery (a) and laparoscopic sacrocolpopexy (LSC) (b)

As shown in Fig. 2, according to the Clavien-Dindo grading system, no patient was classified as grade 4 or worse, which means no reported complications were life-threatening. In TVM surgery, 75.15% (*n* = 124) patients were classified as grade 3a or 3b, who required surgical intervention for complications. This percentage in LSC surgery was 70.68% (*n* = 54). No significant difference was found in Clavien-Dindo classification between TVM and LSC (*p* = 0.090).

**Fig. 3:**
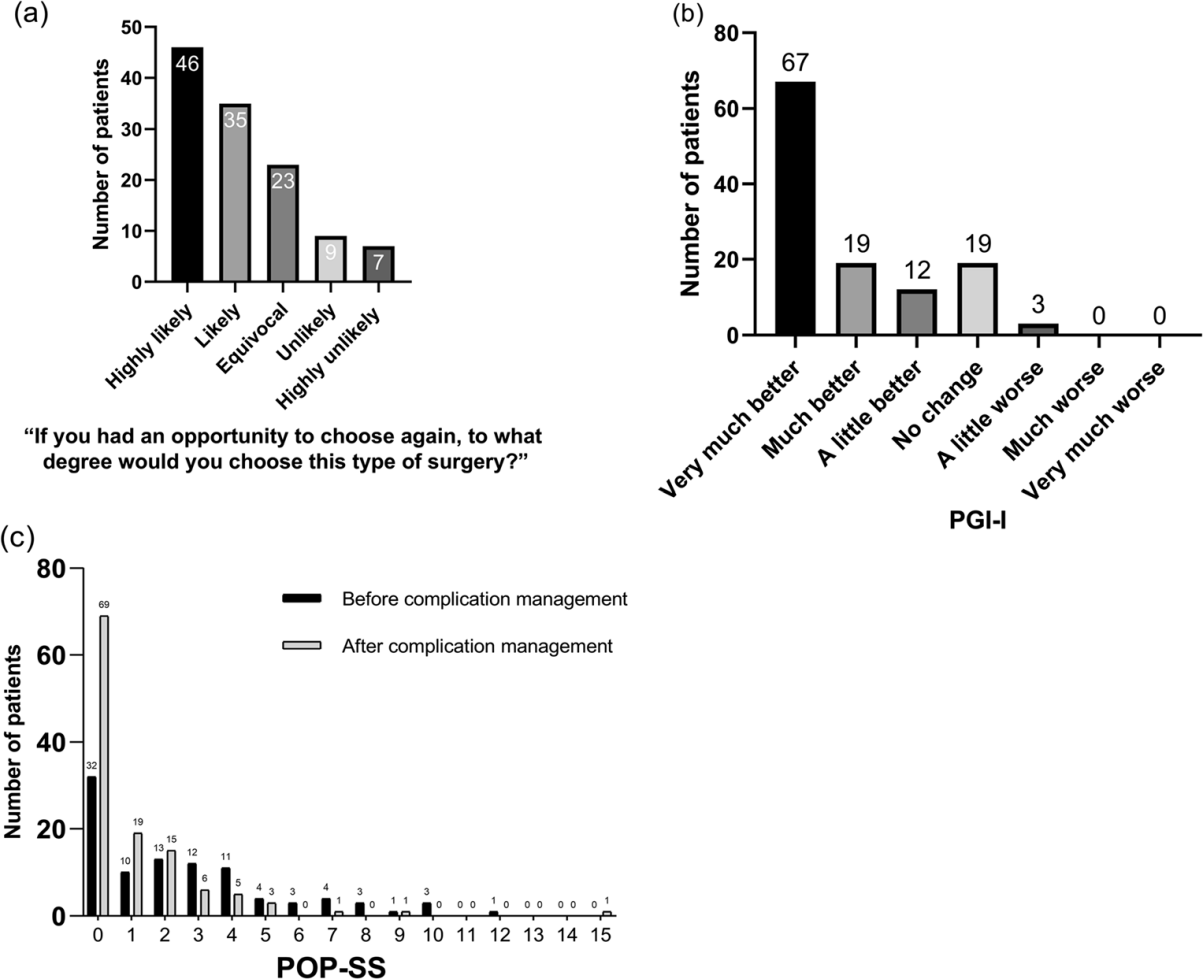
(a) The 5-point Likert-type scale evaluation of surgery choice decisions after TVM surgery. (b) Patient Global Impression of Improvement (PGI-I) scale assessment of the improvement of pelvic organ prolapse symptoms after management for TVM surgery complications. (c) Pelvic Organ Prolapse Symptom Score (POP-SS) assessment before and after management for TVM surgery complications

In the TVM group, the median delay between complication management and the last follow-up was 21.63 (range 7.8–33.77) months. One hundred twenty patients who received TVM completed the 5-point Likert-type scale, PFIQ-7, POP-SS, and PGI-I at the last follow-up. Using the 5-point Likert-type scale to evaluate how much the patients would choose the surgery again, only 16 (13.3%) selected “unlikely” and “highly unlikely” (Fig. 3a). Eighty-one patients (67.5%) selected “likely” and “highly likely.” The remaining 23 (9.2%) selected “equivocal.”

The median PFIQ-7 scores before and after complication management were 38.1 (range 0–142.86) and 4.76 (0–114.28), respectively. PFIQ-7 score was significantly lower after complication management (*p* < 0.001). On the PGI-I scale (Fig. 3b), only 3 patients selected “a little worse;” 19 selected “no change;” 98 out of 120 patients (81.7%) felt better after complication management. Before and after complication management, the median POP-SS was 2 (range 0–12) and 0 (range 0–15), respectively (Fig. 3c). Therefore, there was a significant improvement of symptoms after complication management (*p* < 0.001).

In the management of TVM exposure (Table 3), 65 out of 116 patients received surgical management (including revision or partial excision and complete excision). Compared to category 3 (vaginal mesh exposure > 1 cm), more patients in category 2 (vaginal mesh exposure ≤ 1 cm) received conservative management including observation and office-based trimming (*p* = 0.031). However, management in both category 2 and 3 achieved good satisfaction. There was no difference between category 2 and category 3 in terms of Likert scale (*p* = 0.207) and PGI-I (*p* = 0.646). Both POP-SS and PFIQ-7 were significantly improved in category 2 (*p* = 0.001 and *p* < 0.001, respectively) and 3 (*p* < 0.001 and *p* < 0.001, respectively). In the same way, more patients with asymptomatic vaginal mesh exposure received conservative management compared to symptomatic exposure (*p* = 0.032). No difference was found in terms of Likert scale (*p* = 0.771) and PGI-I (*p* = 0.094). Both POP-SS and PFIQ-7 were significantly improved in asymptomatic (*p* = 0.042 and *p* < 0.001, respectively) and symptomatic exposure (*p* < 0.017 and *p* < 0.001, respectively).

**Table 3:** Management of transvaginal mesh exposure in different complication groups

	Management					Likert scale	PGI-I	POP-SS	PFIQ-7
	Observation	Office-based trimming	Revision or partial excision	Complete excision	*p*	*p*			
Category 2	9	22	18	4	0.031^a^	0.207^b^	0.646^b^	0.001^c^	< 0.001^c^
Category 3	4	16	35	8				0.001^c^	< 0.001^c^
Asymptomatic	5	8	6	0	0.032^a^	0.771^b^	0.094^b^	0.042^c^	0.017^c^
Symptomatic	8	30	47	12				< 0.001^c^	< 0.001^c^

^a^Patient distribution in management types between category 2 and 3 and between asymptomatic and symptomatic complications was compared by Pearson’s chi-squared test

^b^Scale score was compared between category 2 and 3 and between asymptomatic and symptomatic complications by Fisher’s exact test

^c^Scale score before and after complication management was compared in each group by the paired samples Wilcoxon signed rank test

## Discussion

In this study, complications occurred constantly for a long time after synthetic mesh surgery. After management, most patients had a positive attitude about the TVM surgery according to the Likert scale investigation. The PGI-I scale showed that most patients experienced an improvement after management. PFIQ-7 and POP-SS assessments were significantly improved after complication management.

During the performance of the study, there was no official guideline for the management of mesh implantation surgery complications. Complications were managed empirically. However, patient stratification was acceptable. Long-term regular follow-up was vital for the management of TVM complications. Mesh exposure occurred constantly for quite a long time after TVM implantation surgery. Regular follow-up ensured that patients would be treated in a timely manner. In a study by Warembourg et al., the mean interval from TVM implantation to mesh exposure management was 28.1 months [17]. The median length of this interval was 24 (range 5–96) months in a study by Crosby et al. [18]. Marcus et al. also concluded that mesh-related complications could frequently occur over 2 years after the primary operation [19]. In this study, some patients seeking complication management received TVM implantation even over 12 years ago, when TVM surgery just started to be widely used clinically in China. Therefore, patients who had no mesh complications several years after TVM surgery should not be omitted from long-term follow-up.

Patients with asymptomatic vaginal mesh exposure tended to receive more conservative management in this study. Deffieux et al. followed nine women with a median delay of 10 years. They also found that asymptomatic vaginal mesh exposure was feasible for conservative management [20]. We found that category 2 complications were more likely to improve when receiving conservative management compared to category 3. The NICE guidance also recommended that vaginal mesh exposure < 1 cm^2^ could first be treated with non-surgical treatment [21]. However, conservative management could be unsuccessful. In a study by Myrthe et al., 63% patients received conservative management before mesh excision. Abbott et al. found that 59.3% patients need surgical intervention after an initially conservative management [22]. Because patients might experience severe surgery complications when receiving reintervention and experiencing recurrence of POP after reintervention [23], conservative management was an option for specific patients not willing to take the risk of reintervention adverse events. However, patients must be fully informed of the risk of failure.

Few patients (16.36%) in this study experienced referral. Our referral rate was much lower than the reported 47.8% [17]. The reason might be that TVM surgery for POP was mostly conducted in tertiary hospitals in China. There has also been insufficient evidence on the indication for referral so far [24].

We coded all complications successfully using the CTS system. CTS system is an objective method and had guiding significance in clinical practice. However, there are still limitations to the the CTS system. First, this system contains too much terms, which made it difficult to code retrospectively. Batalden et al. found that one third of mesh erosions could not be retrospectively coded because of lacking relevant information [25]. Second, this system is not patient centered. Symptoms are not specifically described and the quality of life not assessed. Therefore, we had to introduce scales as a supplement to assess symptoms and patient satisfaction after management. The combination of the CTS system and quality of life scales contributed to a more comprehensive evaluation of the effectiveness of complication management. Carter et al. conducted a systematic review of the management of mesh complications following surgery for stress urinary incontinence or POP [24]. However, they found that only one study reported the health-related quality of life. In the study by Hokenstad et al., the health-related quality of life after surgical treatment of complications from TVM was evaluated [26]. Of the 41 responders, 54% selected very much better or much better in the PGI-I after complication management, which was 70.83% in this study. Short Form 12 (SF-12) (mental) was significantly improved; however, the Pelvic Floor Distress Inventory-Short Form 20 (PFDI-SF20) was not significantly improved; it comprises three subscales: Urinary Distress Inventory 6, Colorectal-Anal Distress Inventory 8, and Pelvic Organ Prolapse Distress Inventory 6. In both the study by Hokenstad et al. and ours, although we adopted different scales, we had the same idea that evaluation of management by scales should consist of two parts: patient impression (PGI-I, Likert-type scale, and SF-12) and quality of life for POP patients (PFIQ-7, POP-SS, and PFDI-SF20).

Although we emphasized the long-term follow-up after synthetic mesh implantation surgery for POP, different patients were at different risk levels for occurrence of TVM complications. A risk-stratified strategy should be established in the future to perform an efficient long-term follow-up. For the absence of assessment of quality of life, the CTS system seemed to be insufficient for evaluating complications. Further research should be conducted on how to evaluate complications comprehensively.

The strengths of this study are as follows. First, we had specific outcomes for the managements. The scales used in this study revealed the patients’ attitude about POP surgery and complication management, which should be one of the main concerns when we manage the complications. PGI-I revealed the patients’ general impression of complication management. The 5-point Likert-type scale reflected the patients’ impression of the benefit and risk of the operation. Though PFIQ-7 was not designed to evaluate complication management, it described the quality of life as affected by pelvic floor disorders (PFDs). Complications indeed affected the quality of life as other PFDs and PFIQ-7 showed an improvement after complication management in this study. Because these complications were caused by POP surgeries, it was essential that we used POP-SS to assess POP symptoms and whether the POP symptoms were affected by the complication management. Second, we focused on TVM complications and had a relatively large sample size. Previous studies concerning TVM complication management usually had a small sample size ranging from 9 to 148 [17–20, 22, 27]. Third, since this was a study of case series reports, it allowed us to trace patients who underwent surgeries many years ago. The post-surgery delay was not limited by the follow-up time, which was usually just a few years.

There was also a limitation in this study. This was a multicenter complication registry study; only surgeries with complications were reported. Therefore, we could only investigate the characteristics of complications and management of complications. Because of the lack of the total number of all types of POP surgeries, the rate of complication occurrence, risk factors, and potential prevention measures could not be investigated.

## Conclusions

The occurrence of complications related to transvaginal mesh surgery and laparoscopic sacrocolpopexy had a longer post-surgery delay than native tissue repair surgery. Long-term regular follow-up was vital to complication management. Patient satisfaction with the management of transvaginal mesh complications was acceptable. The symptoms and quality of life quantified by scales were significantly improved after complication management.
